# Determinants of Household Catastrophic Health Expenditure: A Systematic Review

**DOI:** 10.21315/mjms2019.26.1.3

**Published:** 2019-02-28

**Authors:** Meram Azzani, April Camilla Roslani, Tin Tin Su

**Affiliations:** 1 Community Medicine Department, Faculty of Medicine & Biomedical Sciences, MAHSA University, Saujana Putra Campus, 42610 Jenjarom, Selangor, Malaysia; 2 University of Malaya Cancer Research Institute (UMCRI), Faculty of Medicine, University of Malaya, 50603 Kuala Lumpur, Malaysia; 3 Department of Surgery, Faculty of Medicine, University of Malaya, 50603 Kuala Lumpur, Malaysia; 4 Centre for Population Health (CePH), Department of Social and Preventive Medicine, Faculty of Medicine, University of Malaya, 50603 Kuala Lumpur, Malaysia; 5 South East Asia Community Observatory (SEACO), Jeffrey Cheah School of Medicine and Health Sciences, Monash University Malaysia, 47500 Bandar Sunway, Selangor, Malaysia

**Keywords:** catastrophic illness, health expenditure, socioeconomic factors, worldwide, systematic review

## Abstract

The World Health Organization estimates that annually 150 million people experience severe (catastrophic) financial difficulties as a result of healthcare payments. Therefore, a systematic review was carried out to identify the determinants of household catastrophic health expenditure (CHE) in low-to high-income countries around the world. Both electronic and manual searches were conducted. The main outcome of interest was the determinants of CHE due to healthcare payments. Thirty eight studies met the inclusion criteria for review. The analysis revealed that household economic status, incidence of hospitalisation, presence of an elderly or disabled household member in the family, and presence of a family member with a chronic illness were the common significant factors associated with household CHE. The crucial finding of the current study is that socioeconomic inequality plays an important role in the incidence of CHE all over the world, where low-income households are at high risk of financial hardship from healthcare payments. This suggests that healthcare financing policies should be revised in order to narrow the gap in socioeconomic inequality and social safety nets should be implemented and strengthened for people who have a high need for health care.

## Introduction

A World Health Organization (WHO) report in 2000 noted that one of the principle roles of the healthcare system is to provide equitable financing, which can protect people from experiencing financial hardship incurred due to the treatment of their illness (1). In 2010, a WHO report highlighted that universal health coverage (UHC) would be enable everybody to access health services without facing financial hardship (2). The majority of the population in low-income countries, and some in middle-income countries, still have to make out-of-pocket (OOP) payments, which is the least sustainable option and an inequitable way of financing health care (3).

WHO estimates that annually 150 million people experience severe (catastrophic) financial difficulties and about 100 million become poor as a result of healthcare payments (2). Some people who cannot afford the necessary health care engage in alternative coping strategies such as borrowing money, mortgaging or selling assets, selling livestock, and withdrawing their children from school. Some even decide not to seek the health care they need, which may lead to deterioration in health as well as reduced productivity and income (4–8).

Previously, it was generally agreed that if a household spent 10% or more of its income on healthcare services, the expenditure was classed as catastrophic (9, 10). In 2003, another measurement for catastrophic health expenditure (CHE) was proposed that uses the household’s capacity to pay and sets the threshold as equal or more than 40% of capacity to pay (11). A later study recommended that the capacity to pay should be refined by considering effective income (measured by total expenditure) minus basic subsistence needs adjusted for household size (11).

Several papers have been published on household CHE across the globe. However, different researchers use different measurements of CHE and cut-off points to identify the prevalence of CHE and associated factors. So far, no systematic review has been conducted on these works to identify the key determinants of CHE among households. Therefore, in order to fill this gap in knowledge, a systematic review was carried out to attempt to identify and summarise the significant factors and household characteristics that are associated with the risk of experiencing CHE.

## Method

The Preferred Reporting Items for Systematic Reviews and Meta-Analyses (PRISMA) guidelines were followed in conducting this systematic review.

### Search Strategy

Both electronic and manual searches were performed. Electronic resources included databases and web pages. The databases searched were PubMed, Medline, ScienceDirect, Web of Science, the Cochrane Library, Embase, CINAHLComplet@EBSCOhost and Scopus. Web pages such as Google Scholar were also searched for non-peer reviewed articles in order to reduce the risk of publication bias. In addition, a manual search of the indices of the included studies was done. The papers that were initially selected ranged in date of publication from January 1986 and July 2016 because most of the publications were released from 1986 onwards. The first to study the CHE determinant was from US, he defined a household as having CHE if OOP was equal or more than 10% of total income (12).

The key words used in the search were catastrophic illness, catastrophic health expenditure, determinants, health expenditure, household factors, financial burden, out of pocket payment and their synonyms.

For a study to be included in this review, the selected study had to define a household as having CHE if OOP was equal to or exceeded 40% of the household’s capacity to pay (11), or had to define it as where OOP was equal or more than 10% of total income (9, 10). The factors associated with CHE that were considered in our review included the household’s socioeconomic characteristics, the socio-demographic characteristics of the head of household, the type of illness and treatment and other illness and treatment related factors, and factors such as land ownership.

### Selection of Studies and Data Extraction

**Figure 1:**
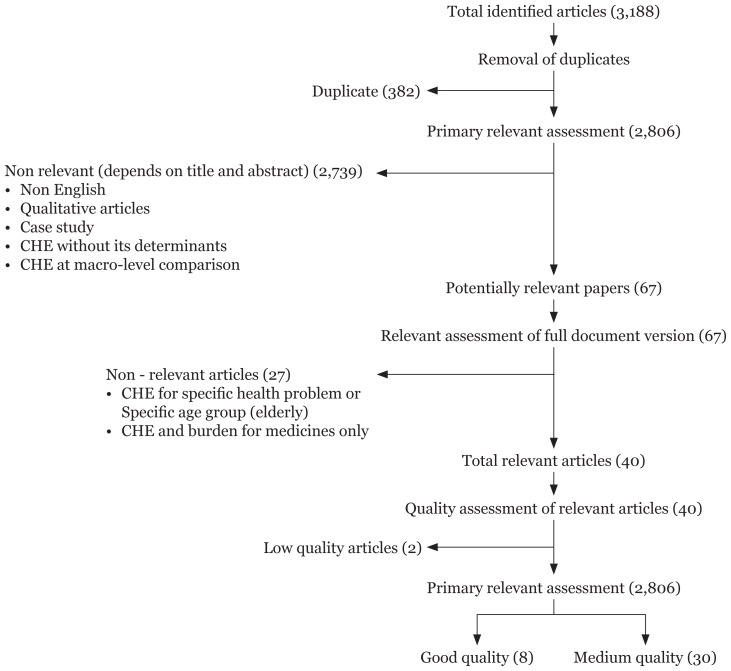
Identification of studies

The selection of articles/papers was conducted in three steps. All searches were carried out by one reviewer and checked by another. The details of all the selected studies were saved in EndNote X6 software, which was also used to screen for duplicate studies. In the first step, the articles were independently selected by two reviewers based on the title and abstract after excluding the duplicate articles in the list. Disagreement on article selection between reviewers was resolved by consensus and consultation with a third reviewer. A total of 3,188 articles were identified, of which 382 were removed because of duplication. Then, we reviewed the titles and abstracts and excluded studies if they reported on CHE without including its determinants or if they reported on the economic burden of households but did not measure CHE. Papers not written in the English language, qualitative studies, case studies, and studies on specific health problems, on specific age group (elderly) and on macrolevel comparisons were also excluded. At this point, 67 papers were eligible for full document review. After evaluating the documents in full, 27 more papers were removed because the CHE only focused on a specific health problem or purchased medication. Out of the remaining 40 studies, 38 were finally selected for this systematic review after quality assessment (Figure 1).

### Quality Assessment

The quality of the initially selected studies was assessed by using the critical appraisal checklist for survey-based studies (13), which comprises the following questions:

1. Did the study address a clearly focused issues/question?
2. Is the research method (study design) appropriate for answering the research question?
3. Is the method of selection of the subjects (employees, teams, divisions, organisations) clearly described?
4. Could the way the sample was obtained introduce (selection) bias?
5. Was the sample of subjects representative with regard to the population to which the findings will be referred?
6. Was the sample size based on pre-study considerations of statistical power?
7. Was a satisfactory response rate achieved?
8. Are the measurements (questionnaires) likely to be valid and reliable?
9. Was the statistical significance assessed?
10. Are confidence intervals given for the main results?
11. Could there be confounding factors that haven’t been accounted for?
12. Can the results be applied to your organisation?

Each criterion was scaled as either reported or not reported. Each question is scored 1 if reported and 0 if not reported. Two reviewers scored the papers independently; the third was involved in arbitration if a disagreement occurred. The final selection was done based on the result of this quality assessment. Only studies of good (7–12 points) or medium (4–6 points) quality were included in this systematic review. Two studies got less than 4 points score, for that, they were considered as low quality and we didn’t include them in this review. The results of the quality assessment are shown in Table 1.

Table 1 provides details of the studies that were selected for review including year of publication, country under study, study design, sample size, outcome measures, method of analysis, results and conclusion.

## Results

### Description of Studies

The final set of 44 selected papers (eight high quality and 30 medium quality) were grouped by country under study into low, middle and high income according to the World Bank classification (14). Thus, four papers investigated CHE in high-income countries (USA, the Czech Republic, South Korea and Portugal), 31 examined the issue in middle-income countries (one each on Colombia, Georgia, Vietnam, Serbia, Bangladesh, West Bank and Gaza (Palestine), Zambia, two on Myanmar, two on Brazil, two on Kenya, three on Iran, three on Turkey, two on Thailand, three on India and seven on China) and three presented research on CHE in low-income countries (Uganda, Burkina Faso and Tanzania) (see Table 2).

### Determinants of Catastrophic Health Expenditure

Table 2 shows the determinants of CHE according to the income level of the country.

#### Household

- Residence
  Eighteen studies examined household residence as one of the potential determinants of CHE (15–32). Six studies found that this variable is not significant (15, 18, 19, 23, 30, 31), but most of the studies found that living in a rural area is a risk factor for incurring CHE (16, 17, 20–22, 24–29). Conversely, one study found that living in a rural area is a protective factor for incurring CHE (32).
- Family size
  A total of 25 studies investigated the relationship between family size and incurring CHE (15, 16, 18–22, 24–28, 31–43). Five studies found that the relationship is not significant (16, 19, 36, 38, 41), ten studies found that a large family size is a risk factor (18, 20, 22, 24, 26, 31, 32, 34, 35, 40) and ten found that a small family size is associated with a higher risk (15, 21, 25, 27, 28, 33, 37, 39, 42, 43).
- Presence of an elderly person in family
  The presence of an elderly person in the household was found to be significant as a risk factor for CHE in all 19 papers that evaluated this variable in their analyses (15–17, 19, 21, 22, 25–28, 32–34, 36, 39, 41–44).
- Presence of children aged under 5 years old
  Of the 16 papers that studied this factor, six found that the presence of children aged under 5 years old is not a significant factor in determining CHE (19, 36, 41, 42, 44, 45), while three studies reported that it is a protective factor (16, 21, 23). The other seven studies found that the presence of children under 5 years old is a risk factor for CHE (22, 25, 26, 28, 32, 34, 39).
- Economic status
  Of the 33 studies that considered the economic status of the household (12, 15, 16, 18–30, 32, 33, 35–37, 39, 41–51), 28 found that households in the lowest expenditure quintile or quartile or lowest income are at higher risk of experiencing CHE. However, five studies reported that households in the highest expenditure quintile/tertile spend more on health care at a level exceeding the capacity to pay threshold (19, 25, 26, 28, 45). The majority of studies expressed economic status in terms of expenditure quintiles (15, 16, 19–22, 24, 25, 30, 35–39, 42, 44, 47–51); however, one of the studies used expenditure quartiles (18) and one of the studies used expenditure tertiles (45). In addition, five studies used income as a continuous variable to express the economic status of the household (12, 23, 27, 29, 33).

#### Head of household

- Gender
  The gender of the head of household was found to be a non-significant factor in 16 papers out of the 27 that analysed this factor (15, 16, 18–20, 24, 25, 30–32, 34, 36, 37, 40, 42, 45). However, in the remaining 11 studies, a female head of household is identified as a risk factor (17, 21, 23, 26–29, 39, 41, 43, 44). In Uganda, this variable was found to be a risk factor among the non-poor, but not significant among the poor (17). In Portugal, it was found to be a risk factor in the year 2005 but a protective factor in the year 2000 (27).
- Age
  The influence of the age of the head of household was analysed in 12 studies. Five studies found that the age of the head of household is not significant (19, 24, 31, 40, 43), whereas seven found that this factor is a significant determinant of CHE, where the older the age of the head of household the greater the risk of experiencing a financial catastrophe (12, 23, 27, 30, 34, 37, 45).
- Employment status
  Of the 17 papers that analysed the employment status of the head of household, 11 found that an unemployed head of household is a risk factor for CHE (12, 16, 19–21, 27–29, 33, 39, 40), while one study done in Colombia reported that being self-employed and a government worker are also risk factors for incurring CHE (37). Another study done in Kenya reported that being self-employed and a government worker are protective factors for incurring CHE (45). One study done in South Korea reported that people who experienced changes in job status from employed to unemployed or were unemployed with no status change were more likely to incur CHE (43). Three studies found this variable is not significant (24, 26, 30) for CHE.
- Educational status
  A total of 22 articles considered educational status as a factor in CHE (15–19, 21–24, 26–31, 34, 39, 40, 42, 43, 50, 51). Six found this factor to be insignificant (18, 30, 34, 40, 42, 43), while 15 papers reported that low education is related to the increased probability of experiencing CHE. The remaining one paper reported that medium education is related to increase the probability of experiencing CHE (31).

#### Illness and treatment

- Hospitalisation (inpatient care)
  Twenty one studies investigated hospitalisation as a possible determinant of CHE (12, 15–17, 19–22, 26, 31, 33, 35–39, 41, 42, 48–50). Hospitalisation was found to be a significant positive predictor of CHE in all but one of the 21 studies.
- Presence of a disabled person
  Of the eleven studies that examined disability as one of the predictors of CHE (16, 18, 19, 26–8, 36, 37, 40, 41, 43), two reported a non-significant relationship (18, 37), while the remaining nine reported a significant association that increases the risk of CHE.
- Presence of a family member with a chronic illness
  The presence of a family member with a chronic illness was found to be a significant factor for CHE in all 17 studies that analysed this factor (15, 18–20, 22, 24, 28, 32, 33, 39–43, 47, 48, 50).
- Other illness and treatment related factors
  Several studies identified some additional CHE determinants, which are (in no particular order of preference) as follows: Healthcare utilisation in general was found to be a risk factor in studies conducted in Burkina Faso (18), Iran (36), India (38) and Kenya (49). The number of illness episodes was identified as a risk factor for CHE in India (20). Drug consumption was a risk factor in a study done in Iran (35). Perceived health status was identified as a risk factor for incurring CHE in Serbia (24). Having a communicable disease, such as tuberculosis, was found to be a risk factor in China (39). Finally, seeking dental care and rehabilitation services were found to be positive predictors of CHE in Iran (41), and seeking health care from a traditional healer were also found to be positive predictors of CHE in Tanzania (40). Utilisation of outpatient health care was risk factor in rural area of China (42) and it was not significant factor in Myanmar (31).

#### Other factors

Lastly, in one study on India, land ownership and a higher level of education for the female heads of household in rural areas are considered to be protective factors that could reduce CHE (34). In Zambia, distance from health care facility, type of illness and type of health care facility were considered to be factors that could affect the CHE, too (30). However, other factors as number of working adults, type of illness and duration stay in slum area, seeking care from public or private hospital were considered by study done in Kenya (45). Study done in South Korea explored other factors and found that, having a member with depression and being married are also risk factors of CHE, however household with negatives self-rated health and household that benefits from medial aid programme are less likely to incur CHE (43). In Myanmar, the researchers explored other factors as ethnic status, household with pregnant women, the number of educated females, the use of insecticide treated bed nets and self-rated health, and they found only the latter was significant (32).

### Prevalence of Catastrophic Health Expenditure

Four studies, two on high-income countries and two on middle-income countries based the calculation of CHE solely on total income. On the other hand, 27 studies measured CHE based solely on capacity to pay.

Only seven studies used both methods of calculation (total income and capacity to pay) and compared the result on the prevalence of CHE derived from the two different methods. Six of these studies were conducted on middle-income countries and one investigated a low-income country.

Thus a total of 11 studies used total income to calculate CHE (12, 19, 23, 24, 26, 28, 30, 34, 44, 46, 49). Six studies used a cut-off point ranging from 5% to 20% of total income (12, 23, 24, 26, 28, 44), while five used a cut-off point, i.e. 10% of total income. It is difficult to compare the results on the prevalence of CHE reported in these studies not only due to the different cut-off points used in the CHE calculation, but also due to the wide time frame (1986–2016) covered by the included studies.

A total of 34 studies used capacity to pay to calculate CHE. Ten used a cut-off point ranging from 10% to 60%, which again makes it difficult to make a comparison across studies. The other 24 studies used only one cut-off point to calculate CHE.

The seven studies that applied capacity to pay as well as total income to calculate CHE (19, 24, 26, 30, 34, 44, 49) found that CHE prevalence estimated by total income is higher than when estimated by household capacity to pay.

### Role of Insurance in the Occurrence of CHE

The role of insurance was examined in 17 studies (15, 16, 21, 22, 25, 26, 28, 29, 33, 35–37, 39, 43, 44, 46, 47) (Table 3). Nine studies found that insurance schemes helped to reduce the risk of incurring CHE (16, 21, 25, 26, 28, 29, 36, 37, 47). On the other hand, two studies found that having insurance was a risk factor for incurring CHE (33, 44). Four studies reported that insurance does not have a significant effect on the occurrence of CHE (33, 35, 43, 46). Two of the studies on the situation in China both reported that the role of insurance depends on the type of insurance scheme that the household has; urban health insurance was found to be protective whereas rural health insurance was found to be a positive predictor that increases the risk of experiencing CHE (22, 39).

## Discussion

This systematic review found that irrespective of the economic status of the country under study the determinants of CHE were broadly similar. For instance, people living in a rural area and belonging to the lower income quintiles were more likely to experience CHE compared to urban residents and richer people (15, 16, 18, 20, 21, 33, 34, 37, 44, 47, 48). However, definition of rural area from country to country may differ. Therefore the role of living area on CHE may vary. Moreover, in most cases there is a strong relation between rural area and large family size. Therefore living area sometimes may be a confounding factor. In general, socioeconomic status was one of the significant indicators of CHE across countries (52).

The low educational level of the head of household was also found to be significant in determining CHE in several studies (15–17, 19, 21–24, 26–29, 39, 50). Generally, educated people have a greater awareness about health and tend to be more frequent users of preventive services and health care. In addition, they are more likely and able to maintain a regular job and cope with financial expenses, including those for health care. Employment status was also found to be significant in many studies (12, 16, 19–21, 27–29, 33, 39, 40) that found heads of household who are not working or are self-employed are at a greater risk of incurring CHE as they have no regular income. This was supported by another finding identified in this review, namely that households headed by a woman had a higher probability of CHE in high- and middle-income countries (17, 23, 44). However, the gender of the head of household was not identified as a significant factor in the studies on low-income countries. This may be due to the fact that in low-income countries women are usually less educated and not able to ensure a sustainable income. The importance of the education of women head of household in the reduction in the likelihood of CHE is also highlighted in the study conducted in India (20).

The results for the influence of other household and head of household characteristics are varied depending on country and therefore general conclusions cannot be drawn from them. Nevertheless, it can be said that, apart from the above three indicators of socioeconomic inequality (income, education and employment status), hospitalisation, presence of an elderly person, presence of a disabled person and presence of a household member with a chronic illness were found to be consistently significant in all studies and regardless of the economic status of the country.

Hospitalisation (inpatient care) was identified as an important determinant as it consumes a high share of OOP payments made by households and leads to financial hardship (12, 15–17, 19–22, 26, 33, 35–39, 48–50).

The presence of an elderly person in a household also significantly increases the probability of that household incurring CHE. This greater probability is associated with the greater need for, and utilisation of, healthcare services by the older generation compared to younger age groups (23). Although there is an increasing ageing population globally, the healthcare system in low- and middle-income countries still does not have sufficient resources to provide the necessary social safety net for the needs of this group. Similarly, the presence of a disabled person in the household increases the risk of experiencing CHE. A disabled person generally has a higher demand for health care, as identified by several studies. Also, health care for chronic illnesses was also found to be a significant determinant of financial hardship (15, 18–20, 22, 24, 28, 33, 39, 40, 47, 48, 50). Responding to the healthcare demands of people with a chronic illness is a common challenge in many countries (53, 54). Moreover, the pandemic of non-communicable diseases (NCDs) has been a serious wake-up call for the healthcare systems of various countries. As many NCDs are chronic in nature and a major cause of mortality all over the world, there is an urgent need to identify evidence-based innovative strategies for the prevention and control of NCDs, and to strengthen healthcare systems to meet the higher healthcare costs of prolonged treatment. Thus, providing a safety net for vulnerable persons (the elderly, the disabled and those with a chronic illness) and the households of which they are part should be seriously considered as a crucial issue in financing health care and achieving UHC.

The majority of the selected studies used the same econometric models to assess the determinants of CHE. However, several studies used different methods and cut-off points to categorise the presence of CHE in households. Therefore, standardisation of both the method and measures used in studying this issue could enable a more relevant and useful comparison of the data across the globe.

Nevertheless, despite the methodological differences in reporting CHE in the various studies, some key points emerged from this review. Firstly, even in high-income countries, households still incur CHE (55). It is generally assumed that households in low-income countries have a higher risk of having CHE (8). However, the findings of this review contradict this assumption because the prevalence of CHE in low-income countries is not that much different from that in middle-income countries. It is possible that many people in low-income countries choose not to seek health care rather than face the financial hardship associated with healthcare payments. This supposition is supported by the findings reported in the studies on Burkina Faso and Iran, where households that utilise more health care are more likely to incur CHE (18, 36).

Secondly, most of the studies identified CHE based on household capacity to pay (15–22, 24–27, 29, 33–40, 44, 47–50), while a few studies used the ratio of OOP to total household income (12, 19, 23, 24, 26, 28, 34, 44, 46, 49). Thirdly, the studies that used both methods of calculation on the same sample population (19, 24, 26, 34, 44, 49) revealed that the prevalence of CHE with regards to healthcare payments to total income was higher than the prevalence based on capacity to pay. However, there is no universally agreed cut-off point for calculating CHE, so researchers use a cut-off point of their own choosing, which limits comparability between studies. However, the most frequently used cutoff points are 10% of household income and 40% of household capacity to pay.

It is also necessary to reach a consensus on a single method of defining CHE (either based on income or capacity to pay). Hence, to validate these two methods, it would be useful to conduct further studies that define CHE by using both methods as in previous research conducted in Thailand (19), India (34) and Brazil (44). Furthermore, qualitative studies should be undertaken on households by using an in-depth interview approach to identify their perceived financial hardship and coping strategies. An analysis of the interviews would help to identify a method that is both sensitive and rational that can accurately capture the financial catastrophe experienced by households.

Thirdly, the role of insurance was found to vary among the included studies. In China, there are three government insurance schemes, the New Rural Cooperative Medical Scheme for rural residents and the Urban Employee Basic Medical Insurance Scheme and Urban Resident Basic Medical Insurance Scheme for urban residents (21). The prevalence of CHE was found to depend on the type of insurance scheme that the household had; the urban health insurance is protective while the rural one is a positive predictor that increases the risk of CHE. One study reported that enrolment in the rural insurance scheme had no effect on CHE prevalence among households when compared to those without insurance, and a possible explanation for this result is the limited coverage for outpatient, self-treatment and traditional medicine treatment (56).

The difference in the prevalence rate of CHE is not only related to the availability of health insurance, but also to the sampling used in the study design. For instance, in Thailand, the UHC policy consists of three major government schemes: the Civil Servant Medical Benefit Scheme, the Social Security Scheme, and the Universal Coverage Scheme, which provide comprehensive outpatient and inpatient coverage through somewhat different benefit packages (19). If the sample consists only of poor urban households, there is likely to be a high prevalence of CHE even under UHC. One possible explanation for the persistence of CHE among poor households in Thailand even after the implementation of the UHC may be due to the participants in the study seeking care from a facility at which they are not registered because care has to be accessed from facilities that operate under at least one of the three Schemes (19). However, Study in Zambia found reduction of CHE after implantation of user fees exemption policy (30).

Reconsideration and upgrading of the health insurance benefits package is crucial to reach a satisfactory level of financial risk protection. In Portugal for instance, although there is an exemption policy for children, the elderly and unemployed, that exemption does not cover the cost of pharmaceutical products and as such causes high OOP expenditure and financial burden (57). Likewise, in Uganda, the policy of charging users fees for health care was eliminated in 2001 in order to increase healthcare utilisation among the poor. However, the prevalence of CHE did not decrease because prescribed medicines were not made available at public hospitals and patients had to purchase them from private pharmacies (17).

In some low- and middle-income countries such as Bangladesh, Kenya, Tanzania, Myanmar and India, the healthcare financing system remains dependent on OOP expenditure. Such dependency reflects a less sustainable way of financing a healthcare system and as such is unable to guarantee any financial protection for vulnerable users of the healthcare system (20, 40). Similarly, Colombia and Turkey are facing issues in terms of the equity and quality of healthcare services, which ultimately jeopardises both of these countries’ intentions of achieving UHC (26, 37). Also, in both the Czech Republic and Vietnam, the continuous budget deficit weakens the possibility of UHC continuing (23, 58).

In Iran, the existing comprehensive national insurance system requires an expansion of the benefit package and a decrease in the co-payments to secure financial protection and avoid CHE (36). Similarly, in Georgia, both inpatient coverage and drug benefits need to be expanded (48). In Serbia, the health insurance fund does not fully cover all services required and the existence of high informal payments increases the risk of CHE (24).

In Brazil, private health insurance leads to a rise in the risk of CHE by increasing the demand for specialised and costly health services that further escalates the inequality of healthcare utilisation among the population (44). In Burkina Faso, although community-based health insurance has been introduced, coverage remains limited and that’s because it is a voluntary insurance, and there is currently zero possibility of achieving UHC (59). In South Korea, the moderate impact of insurance cover on financial protection shows that people with health insurance are still paying fairly high OOP payments (43). Lastly, in the USA, cost-sharing in Medicare and the high premium for insurance are more likely to expose low-income families to a greater risk of CHE (60).

The measurement of CHE has become increasingly important because it is one of the key parameters for financial protection in achieving UHC. However, this review discovered that there were some general weaknesses in the studies on CHE. For instance, the incidence of chronic illness is based on self-reported data rather than accurate medical diagnosis, which could lead to an over-estimation of the health expenditure on chronic illness. Moreover, payment for health care is solely based on the monetary value and the cost for institutional health care. Thus the methodology cannot capture the in-kind payments that are common in rural areas of low-income countries. Finally, CHE is estimated for households that decided to seek health care, but this does not capture the complete scenario because there are households that choose not to seek health care to avoid the financial hardship that would be incurred in making healthcare payments.

## Study Limitation

For this review, papers published in languages other than English were excluded, so it is possible that some studies were missing. However, in the extensive search of databases only three papers published in non-English languages were found (and excluded). There are many non-English researches on CHE and these can be found in Google scholar. And several studies among them were in-depth studies analysed panel data. There is also a possibility that some studies which have been classified as ‘grey literature’ were missing and thus, there may be some risk of publication bias. However, this is not uncommon limitation for systematic review.

## Conclusion

The crucial finding of this study is that socioeconomic inequality plays an important role in the incidence of CHE all over the world, where low-income households are at high risk of financial hardship from healthcare payments and of falling below the poverty line as a consequence. The educational status and employment status of the head of household, need for healthcare to manage a chronic illness, and the presence of an elderly or disabled household member were also identified as predictors of CHE. Simultaneously, we would like to highlight that the representativeness of data and source of data and data collection system in different studies are different. Therefore the strength of the factors which effects catastrophic health expenditure and impoverishment may vary and may not be similar.

The findings of this review suggest that healthcare financing policies should be revised in order to narrow the gap in socioeconomic inequality and social safety nets should be implemented and strengthened for people who have a high need for health care such as the elderly, people with a chronic illness and disabled persons. Also, CHE can be used as an indicator for comparing fairness in the financing of health care across countries and within a country over a period of time. It can also be used to measure the level and effectiveness of financial protection, which is an important parameter of UHC.

## Figures and Tables

**Table 1:** Data extraction and the quality of the studies

Year of publication	Title	Author	Country	Survey type	Sample size	Variables	Result (outcome)	Representativeness	Quality
**1986**	Families with catastrophic health care expenditures	Wyszewianski (1986)	Michigan, US	1977 National Medical Care Expenditure Survey (NMCES)	14,615 HH*	**Independent variables:** Demographical characteristics, income, poverty status, HH head age and work status. Dependent variables: CHE** and OOP^***^	4.2% of all HH had a CHE where the OOP was ≥ 20% of their total income and 9.6% of the HH had CHE at ≥ 10% threshold. The determinants of CHE were low income where 2/3 of them below the poverty line, HH head age > 65 years old or unemployed HH head.	Representative	Medium
**2006**	Catastrophic household expenditure for health care in a low income society: A study from Nouna district, Burkina Faso	Su et al. (2006)	Burkina Faso	Nouna health district household survey 2000–2001	Sample size was 800 HH, 320 urban, 480 rural, 774 were included in the study.	**Independent variables** HH characteristics (HH residence and economic status), sex and the educational status of the HH head, treatment and illness pattern variables. **Dependent variables** CHE.	CHE = 8.66% (based on ratio of health payment of 40% or more of CTP****) Seeking health care, average number of illness, chronic illness, and economic status were the factors found to be associated with CHE.	Representative Response rate 96%	Good
**2006**	Understanding the impact of eliminating user fees: Utilisation and catastrophic health expenditures in Uganda	Xu et al. (2006)	Uganda	Socioeconomic Surveys of Government of Uganda, 1997, 2000 and 2003	6,655, 10,691 and 9,710 households in turn, comprising 33,988, 53,761 and 47,468 individuals in 1997, 2000 and 2003, respectively.	**Independent variables** Type of provider, presence of a member of 65 years old, presence of a member of less than 5 years old, HH head educational status and sex, HH residence and inpatient service use. **Dependent variables** CHE.	CHE = 2.92% (based on ratio of health payment of 40% or more of CTP) The determinants of CHE: inpatient service used among poor, HH member of > 65 years, HH head with little education, urban settlement was protective for non poor and not for poor, the elimination of fees didn’t reduce the CHE incidence.	Representative	Medium
**2009**	Which households are at risk of catastrophic health spending: Experience in Thailand after universal coverage	Somkotra, Lagrada (2009)	Thailand	Household Socioeconomic Surveys (SES) 2006. And data from the SES in 2000, 2002, and 2004 were also examined.	24,747, 34,785, 34,843 and 22,547 HH collected in 2000, 2002, 2004, 2006, respectively.	**Independent variables:** Sex, age and educational status of the HH head, presence of elderly, presence of children, HH economic status, HH size, insurance coverage, presence of a member with chronic illness or disability, or being hospitalised and the type of health care. **Dependent variables:** CHE.	CHE = 0.77% (2006), 0.97% (2004), 1.07% (2002), 1.23% (2000) (based on ratio of health payment of 40% or more of CTP). Based on health payment of ≥ 10% of total income; the CHE = 4.03% (2006), 4.8% (2004), 5.03% (2002), 6.44% (2000). Important determinants were inpatient care at public providers among the poor, and the outpatient care at private facilities, presence of elderly and members of chronic illness or disability or having a member hospitalised in past 12 months. Higher education HH were with less probability of having CHE.	Representative	Medium
**2009**	Household catastrophic health expenditure: Evidence from Georgia and its policy implications	Gotsadze et al. (2009)	Georgia	Health Care Utilisation and Expenditure survey conducted during May–June 2007	2,859 households	**Independent variables:** Residence, a member of chronic illness, hospitalisation and the HH economic status. **Dependent variables:** CHE.	CHE = 11.7% (based on ratio of health payment of 40% or more of CTP) CHE prevalence was 27 times with those with chronic illness and hospitalisation. The rich were less likely to have CHE.	Representative	Medium
**2010**	Catastrophic health expenditure and impoverishment in Turkey	Yardima et al. (2010)	Turkey	Household Budget Survey, Consumption Expenditures, 2006.	8,558 households	**Independent variables:** Age, sex, educational and employment status of the head of the HH, family size, economic status presence of preschool kids, HH settlement, insurance coverage and presence of a member with disability. **Dependent** Types of OOP and CHE.	CHE = 0.6% (based on ratio of health payment of 40% or more of CTP). Significant factors were HH residence, presence of a member with disability, HH head education status and work status, presence of elderly, presence of preschool children and insurance coverage.	Representative	Medium
**2010**	The influence of the rural health security schemes on health utilisation and household impoverishment in rural China: Data from a household survey of Western and Central China	Shi et al. (2010)	China	Community, household survey 2008 in Hebei and Shaanxi provinces, and the Inner Mongolia Autonomous Region, which represent Western and Central China	3,340 households	**Independent variables:** Age, gender, ethnicity, education level, Occupational status, marital status and religion of the HH head, insurance Status, presence of a member with chronic illness or disability, number of episodes of in-patient visits, unmet inpatient need, HH per capita expenditure, health payment, HH in poverty and household capacity to pay. **Dependent variables:** CHE HH impoverished due to health payment.	The incidence of CHE = 14.3% (based on ratio of health payment of 40% or more of CTP) The CHE determinants were poorer HH, low education of HH head and presence of a member with chronic disease. Insurance found to be reduced the risk of CHE. Those with lower expenditure quintile were more likely to be impoverished (8%).	Representative Response rate 99.8%.	Medium
**2010**	Catastrophic out-of-pocket payment for health care and its impact on households: Experience from West Bengal, India	Mondal et al. (2010)	India	Household survey 2007	3,150 HH, 15,277 individuals	**Independent variables:** Prevalence of illness, HH characteristics; size, residence and the economic status **Dependent variables:** CHE	> 30% of HH spend ≥ 40% of non-food expenditure on inpatient care, those used private hospital spend 25% of their annual income on inpatient care, rural residence, birth delivery, presence of a member with chronic illness, hospitalisation, number of illness episodes, type of medical care were considered as the most important determinants of CHE.	Representative	Medium
**2011**	Study of catastrophic health expenditure in China’s basic health insurance	Zhou, Gao (2011)	China	Forth National Health Service Survey (NHSS) conducted in Shaanxi Province (west) 2008	1,215 households covered by UEMS or URMS (insurance scheme), and 2,875 households covered by NCMS were chosen in this study.	**Independent variables:** Illness, presence of a member with chronic disease, outpatient and inpatient used, sex and the education level of HH head, presence of a member with 65 years old, location of HH, family size, economic status and the insurance type **Dependent variables** CHE	CHE=16.87%–19.62% (based on ratio of health payment of 40% or more of CTP) The important determinants were presence of elderly, hospitalisation, poor health, presence of a member with chronic illness, family size and the HH economic status.	Representative	Medium
**2011**	Determining factors of catastrophic health spending in Bogota, Colombia	Amaya, Ruiz (2011)	Colombia	Expenditure Survey performed by Cendix (2001)	2,810 households	**Independent variables:** Age, gender, the work status and social security of the HH head. HH income in different quintiles, HH size, disability, child births, **Dependent variables:** CHE	CHE at ≥ 20% of CTP was 4.5%, it was higher among low income HH. The significant risk factors were absence of social security and having inpatient admission, and those with small family size and when the HH head was > 60 years old or have no work	Representative	Medium
**2011**	Effect of household and village characteristics on financial catastrophe and impoverishment due to health care spending in Western and Central Rural China: A multilevel analysis	Shi et al. (2011)	China	A cross-sectional community household survey 2008	A total of 3,334 residents from 3,340 households	**Independent variables** Age, sex, race, marital status, education, occupation and religion of HH head, no. of patient with chronic illness, hospitalisation, HH insurance status, HH income, adult illiteracy rate, availability of health clinic, distance from village to clinic, and to county hospital. **Dependent variables:** CHE Impoverishment	CHE = 18.4% (based on ratio of health payment of 40% or more of CTP) Households with low per capita income, having elderly, hospitalised or chronically ill members, and whose head was unemployed were more likely to incur financial catastrophe and impoverishment due to health expenditure. Both catastrophic and impoverishing health payments increased with increased village deprivation.	Rural only response rate of 99.8%	Good
**2011**	Catastrophic spending on health care in Brazil: Private health insurance does not seem to be the solution	Barros et al. (2011)	Brazil	2002–2003 Brazilian Household Budget Survey	37,830 urban households only	**Independent variables:** Health expenses (medicine), insurance, HH head sex, presence of elderly and HH economic status**Dependent variables:** CHE	CHE = 2% at 40% CTP, and 15.5% according to 10% of total income. Poorest had seven times greater risk of CHE than the rich, Socioeconomic position, sex of the head is insignificant, and presence of elderly increase the risk, HH with health insurance at greater risk of CHE.	Used only urban HH	Medium
**2012**	Unexpected impact of changes in OOP payments for health care on Czech household budgets	Krutilova, Yaya (2012)	Czech	Household budget survey, 2007, 2008 and 2009.	3,000 households, 2007, 2008 and 2009. Before and after user fees	**Independent variables:** Sociodemographic factors (head age, sex, educational status and work status, residence, economic status, no. of kids. **Dependent variables:** Types of OOP CHE	CHE = 11.89% (based on 5% or above of total income). Most affected HH were those with pensioners, elderly and low income.	Representative	Medium
**2012**	Factors affecting catastrophic health expenditure and impoverishment from medical expenses in China: Policy implications of universal health insurance	Li et al. (2012)	China	Fourth National Health Service Survey (NHSS, 2008).	55,556 households	**Independent variables** Sex, educational and employment status of HH head, health insurance status, HH economic status, HH size, having at least one member older than 60 or younger than 5 years or with tuberculosis or any chronic non-communicable condition or hospitalised member. **Dependent variables**: CHE	CHE = 13% (based on ratio of health payment of 40% or more of CTP) Determinants of CHE were HH headed by a female, an unemployed person or having little education. Having at least one member who was elderly, ill from tuberculosis or chronic non-communicable illness, or hospitalised, without insurance and rural HH were at greater risk of CHE.	Representative	Good
**2012**	Measuring incidence of catastrophic OOP health expenditure: With application to India	Pal (2012)	India	Household Consumer Expenditure Survey 2004–2005	Not mentioned	**Independent variables:** Economic variables: land, wealth index, regular salary, education status, plus the socioeconomic factors as HH size, No. of children and elderly, sex of HH head and his age. **Dependent variables:** CHE	CHE = 14.68% among the poorest and 34.90% among the richest (using 10% threshold of total budget) CHE = 4.84% among poor −13.76% among rich (based on 40% threshold of CTP) Large HH, presence of children and elderly and aged HH head were the significant determinants of CHE.	Representative	Medium
**2012**	Inequality in HH catastrophic health care expenditure in a low-income society of Iran	Kavosi et al. (2012)	Iran	WHO survey in 2003 and repeated again by research team in 2008	1123 households in 2003, 635 households in 2008	**Independent variables:** HH head sex, HH size, presence of a member of > 65 years old or less than 5 years old, HH insurance status, presence of a member with disability, HH economic status, using dentistry service or inpatient or out patient service. **Dependent variables:** CHE	CHE = 12.6% in 2003, 11.8% in 2008 (based on ratio of health payment of 40% or more of CTP) The important determinants were presence of HH member over 65 years old or with disability, lower economic quintile, using of inpatient, outpatient and dentistry health services and lack of insurance.	Representative	Medium
**2012**	Iranian household financial protection against catastrophic health care expenditures	Moghadam et al. (2012)	Iran	Iranian household survey 2008	39,088 households	**Independent variables:** HH economic status, family size, inpatient and outpatient health care utilisation, drug consumption, drug addiction cessation and insurance status. **Dependent variables:** CHE	CHE = 2.8% (based on 40% of CTP) Important determinants were large family size, low economic status, inpatient and outpatient health care utilisation, drug consumption, drug addiction cessation	Representative	Medium
**2012**	Catastrophic health care spending and impoverishment in Kenya	Chuma, Maina (2012)	Kenya	Health expenditure and utilisation survey, 2007	8,414 households	**Independent variables:** HH economic status, Type of health care utilisation **Dependent variables:** CHE Impoverishment	CHE = 15.5% (using 10% threshold of total budget) and 11.4% (based on ratio of health payment of 40% or more of CTP). Lower income HH was more likely to had CHE. The use of outpatient services leads to CHE more than the use of inpatient services. The poverty level = 54.9% and it increased 2.7% after health care payment.	Representative	Medium
**2012**	Measuring the catastrophic and impoverishing effect of household health care spending in Serbia	Arsenijevic et al. (2012)	Serbia	Serbian Living Standard Measurement Study (LSMS)	5,557 households	**Independent variables:** HH economic status, residence, HH size, HH head educational level, age, employment, marriage status, gender and presence of member with chronic illness **Dependent variables:** CHE Impoverishment	CHE = 2%–2.4% (based on total income) and 0.8%–1.1% (base on CTP), significant determinants were rural residence, not married HH head, low education, low economic status, large family size, presence of member with chronic illness	Representative	Medium
**2013**	Financial burden of HH OOP health expenditure in Vietnam: Findings from the National Living Standard Survey 2002–2010	Van Minh et al. (2013)	Vietnam	Vietnam Living Standard Survey 2002, 2004, 2006, 2008 and 2010	45,000, 37,200, 36,756, 36,756 and 46,995 households in 2002, 2004, 2006, 2008 and 2010, respectively	**Independent variables:** HH head sex, HH size, presence of a member of > 65 years or less than six years, HH insurance status, HH economic status and residence **Dependent variables:** CHE Impoverishment	CHE = 4.7% in 2002, 5.7% in 2004, 5.1% in 2006, 5.5% in 2008 and 3.9% in 2010 (based on ratio of health payment of 40% or more of CTP) The important determinants were presence of HH member over 65 years or less than six years, higher economic quintile and living in rural area. Those who pushed into poverty were 3.4%, 4.1%, 3.1%, 3.5%, 2.5% in 2002, 2004, 2006, 2008 and 2010, respectively.	Representative	Good
**2013**	Catastrophic health expenditure and entitlement to health services in the occupied Palestinian territory: A retrospective analysis	Ashour et al. (2013)	West bank and Gaza (Palestine)	Palestinian Consumption and Expenditure Survey, 2010	3,754 households	**Independent variables:** HH head sex, education and work status. HH income, residence and insurance status. **Dependent variables:** CHE	CHE = 2.4% (based on ratio of health payment of 40% or more of CTP). The prevalence was less among insured HH in compare to uninsured ones. CHE significantly differed according to different factors considered (HH head sex, education and work status. HH income and residence)	Representative	Medium
**2013**	Health-Related financial catastrophe, inequality and chronic illness in Bangladesh	Rahman et al. (2013)	Bangladesh	Household survey of 1600 households in Rajshahi city August to November 2011	1,600 households	**Independent variables:** HH head sex and educational level, presence of a member of > 65 years, HH economic status and type of health care utilised. **Dependent variables:** CHE	CHE = 9% (based on ratio of health payment of 40% or more of CTP). The important determinants were presence of HH member hospitalised or had a chronic illness, number of illness, the economic status and the educational level of the HH head.	Represented only the urban household Response rate 99.6%	Medium
**2013**	Assessing the magnitude, distribution and determinants of catastrophic health expenditure in urban Lucknow, North India	Misra et al. (2013)	India	Household survey in 2011–2012 in urban Lucknow	400 households	**Independent variables:** HH economic status, HH size and type of health care utilised. **Dependent variables:** CHE	CHE = 11.5%, 4%, 3%, 2.75% at 10%, 20%, 30% and 40% of HH capacity to pay, respectively. Important determinants were outpatient and inpatient health care utilisation and the economic status of the HH.	Urban representation	Medium
**2013**	Catastrophic health expenditure in un urban city: Seven years after universal coverage policy in Thailand	Weraphong et al. (2013)	Thailand	A cross sectional survey in Nakhon Sawan Municipality in 2008	406 sampled households	**Independent variables:** HH economic status and type of health care utilised the cost components of treatment and the insurance scheme. **Dependent variables:** CHE	CHE = 7.1% in non-poor and 12.5% poor (based on 10% of total HH income). Important determinants were the use of public and private hospitals and clinics, transportation cost, loss of time cost and civil servants card holder.	Urban representation	Medium
**2013**	Household catastrophic medical expenses in Eastern China: Determinants and policy implications	Li et al. (2013)	China	Health care utilisation and expense survey, 2008	11,577 households	**Independent variables:** HH economic status, residence, HH size, presence of children or elderly. HH head educational level, presence of a member with chronic illness or being hospitalised and the insurance scheme. **Dependent variables:** CHE	CHE = 9.24% to 24.79% (based on ratio of health payment of 40% or more of CTP). Important determinants were low economic status, rural residence, hospitalisation, member with chronic illness, presence of elderly or children, large HH size, no or low education of HH head and type of insurance scheme.	Representative	Medium
**2014**	Catastrophic health expenditure and rural household impoverishment in China: What role does the new cooperative health insurance scheme play?	Li et al. (2014)	China	Fourth National Health Service Survey (NHSS, 2008)	56,400 households	**Independent variables:** HH economic status, presence of elderly or children, HH size, HH head sex, work status and educational level, hospitalisation and presence of a member with chronic illness, insurance status. **Dependent variables:** CHE Impoverishment	CHE = 14.4% (based on ratio of health payment of 40% or more of CTP), poverty = 9.2%. Important determinants were hospitalisation, member with chronic illness, presence of elderly or children, HH head female, no or low education and unemployment of the HH head and type of insurance scheme.	Representative	Medium
**2014**	Correlates of out-of-pocket and catastrophic health expenditures in Tanzania: Results from a national household survey	Brinda et al. (2014)	Tanzania	National Panel Survey (TZNPS) in 2008–2009	3,265 households	**Independent variables:** HH size, HH head age, sex, work status and educational level, presence of a member with chronic illness or disability. **Dependent variables:** CHE	CHE = 18% (based on ratio of health payment of 40% or more of CTP). Significant determinants were large HH size, unemployment or manual labourer HH head, presence of a member with chronic illness or disability.	Representative	Medium
**2014**	Out-of-pocket health care expenditure in Turkey: Analysis of the 2003–2008 household budget surveys	Brown et al. (2014)	Turkey	Turkish Household Budget Surveys (2003–2008)	800 household surveyed per month in all the years except 2003, where 2,200 household surveyed in that year.	**Independent variables:** HH size, economic status, presence of elderly or less than 5 years children, residence, HH head sex, work status and educational level, presence of a member with illness or disability and insurance status. **Dependent variables:** CHE	CHE = 1.2%–17.6% at different years (2003–2008) at different cut off points (2.5%, 5%, 10%, 15% and 20%) of total HH expenditure. Significant determinants were presence of elderly or less than 5 years children, or presence of a member with illness or disability, no insurance and low education of HH head.	Representative	Medium
**2014**	Financial catastrophe and poverty impacts of out-of-pocket health payments in Turkey	Narci et al. (2014)	Turkey	Turkish Household Budget Surveys (2004–2010)	62,886 households in study years	**Independent variables:** HH size, economic status, presence of elderly or less than 5 years children, residence, HH head sex, work status and educational level, presence of a member with disability, inpatient care and insurance status. **Dependent variables:** CHE Impoverishment	CHE varied according to different thresholds used and at different years using both methods (capacity to pay and total income method). All the determinants studies had a positive relationship to CHE except the work status of household head. The prevalence of impoverishment was less than 1 in all the studied years.	Representative	Medium
**2014**	Catastrophic healthcare expenditure – drivers and protection: The Portuguese case	Kronenberg, Barros (2014)	Portugal	Portuguese Household Budget Survey (2000 and 2005)	10, 020 households (2000) 10,403 household (2005 )	**Independent variables:** HH size, economic status, presence of elderly or less than 5 years children, residence, HH head sex, age, work status and educational level, presence of a member with disability **Dependent variables:** CHE Impoverishment	CHE = 5.03%–32.76% at different thresholds in 2000 and 2005 year analysis (based on the CTP calculation). Important determinants were age of HH head, presence of member with disability, economic status and rural residence in 2005.	Representative	Medium
**2014**	Socioeconomic inequality in catastrophic health expenditure in Brazil	Boing (2014)	Brazil	National Household Budget 2002–2003 and 2008–2009	48,470 HH in 2002–2003 and 55,970 HH in 2008–2009	**Independent variables:** HH economic status, HH head level of education **Dependent variables:** CHE Socioeconomic inequality	CHE = 0.7% and 21.0%. CHE prevalence and socioeconomic inequality increased from 2002–2003 to 2008–2009. Determinants: The low economic status and low educational level.	Representative	Medium
**2015**	Measurement and explanation of socioeconomic inequality in catastrophic health care expenditure: Evidence from the rural areas of Shaanxi Province	Xu (2015)	China	National Household Health Service Surveys of Shaanxi Province, 2008 and 2013	3,217 HH in 2008 and 13,085 HH in 2013	**Independent variables:** HH head gender and educational level. HH characteristics (presence of a member of 65 years old, presence of a member of less than 5 years old, economic status, HH size and insurance status. Presence of a member with chronic illness, or receiving inpatient or outpatient care **Dependent variables:** CHE Income-related inequality	CHE = 17.19% in 2008 and 15.83% in 2013, the inequality in facing CHE strongly increased. The determinants of CHE were HH economic status and HH size in 2013, the absence of commercial health insurance and having elderly members	Representative for rural area	Good
**2015**	Catastrophic health expenditure and its determinants in Kenya slum communities	Buigut (2015)	Kenya	Data from Indicator Development for Surveillance of Urban Emergencies (IDSUE) project, 2011–2013	9447HH	**Independent variables:** HH head gender, age and work status, HH characteristics (presence of a member of less than 5 years old, economic status and insurance status. Type of illness, seeking care in case of illness and the type of health care facility **Dependent variables:** CHE	CHE = 1.52%–28.38%. The CHE determinants were the number of working adults in a HH and membership in a social safety net appear to reduce the risk of catastrophic expenditure. Seeking care in a public or private hospital increases the risk of CHE.	Representative for slums	Medium
**2015**	Health care expenditure of households in Magway, Myanmar	Khaing (2015)	Myanmar	Cross-sectional Household survey, 2012	700 HH	**Independent variables:** HH head gender, age and education level, HH characteristics (family size, residence). Seeking outpatient or inpatient health care. **Dependent variables:** CHE	CHE = 25.2% in urban area and 22.7% in rural area. The CHE determinants were HH medium educational level, large family and hospitalisation.	Representative	Medium
**2015**	Financial risks from ill health in Myanmar-Evidence and policy implications	Htet (2015)	Myanmar	World health survey, 2002–2003	6,045 HH	**Independent variables:** HH head gender, female education level, self-rated health. HH characteristics (family size, residence, presence of a member of less than 5 years old or > 60 years old, economic status, presence of pregnant woman, ethnicity, use of insecticide treated bed net). **Dependent variables:** CHE	CHE = 41%. CHE determinants were presence of a member of less than 5 years or > 60 years old, large HH size, poor self-rated health, poor HH, presence of member with chronic illness and being of ethnic minority, female head	Representative	Medium
**2016**	Catastrophic health expenditure according to employment status in South Korea: A population-based panel study	Choi (2016)	South Korea	Korean Welfare Panel Study Survey (KOWEPS), 2009–2012	5,335 HH	**Independent variables:** HH head gender, age, education level, change in employment status, marital status, self-rated health), HH characteristics (family size, HH income, insurance status, presence of a member of > 65 years, with chronic disease or depression or disability). **Dependent variables:** CHE	CHE = 4.1%, The CHE determinants were female HH head, married, change job status, family size of two persons, negative self-rated health, having a member of > 65 years old, or a member with chronic illness, disability or depression	Representative	Good
**2016**	Catastrophic health expenditure after the implementation of health sector evolution plan: A case study in the West of Iran	Piroozi (2016)	Iran	A cross sectional survey in Sanandaj city, 2015	663 households	**Independent variables:** Gender of HH, HH characteristics (presence of a member of 65 years old, presence of a member of less than 5 years old, economic status, HH size, insurance status, receiving dental care, rehabilitation, impatient and outpatient spending). **Dependent variables:** CHE	4.8% of all HH had a CHE. The determinants of CHE were household economic status, presence of elderly or disabled members in the household and utilisation of inpatient or rehabilitation services.	Representative for West of Iran	Good
**2016**	Does user fee removal policy provide financial protection from catastrophic health care payments? Evidence from Zambia	Masiye (2016)	Zambia	Zambia Household Health Expenditure and Utilisation Survey (ZHHEUS) in 2014	12,000 households	**Independent variables:** Gender of patient, HH head age, work status and educational level, HH characteristics (economic status, residence, distance to health care facility). Facility type of health care and type of illness). **Dependent variables:** CHE Extent of financial protection after abolish user fees policy	CHE = 10%, the CHE prevalence reduced after implementation of user fees removal policy. The determinants of CHE were age of patients, distance, facility type, HH economic status and type of illness.	Representative Response rate 99.4%	Good

* HH (household)

** CHE (catastrophic health expenditure)

** OOP (out of pocket payment)

**** CTP (capacity to pay)

**Table 2:** Factors associated with catastrophic health expenditure

No	Income category	Author-year	Country	Household (HH) characteristics					Household head characteristics				Illness and treatment factors		
		
				Residence (Rural)	Family size	Presence of elderly of > 60–65 years	Presence of children under 5	Economic status	Gender	≥60–65 years	Employment status	Level of education	Have a member hospitalised	Presence of disable person	Presence of a member with chronic illness
1	High	Krutilova, Yaya (2012)	Czech	NS	NA	NA	_	+Low	+Female	+	+ Unemployed	+ Low	NS	NA	NA
2	High	Wyszewianski (986)	USA	NA	NA	NA	NA	+ Low	NA	+	+ Unemployed	NA	+	NA	NA
3	High	Kronenberg, Barros (2014)	Portugal	+ (2005)	− Large	+	NA	+Low	− Male (2000) + Male (2005)	+	+ Unemployed	− High	NA	+	NA
4	High	Choi (2016)	South Korea	NA	+Small	+	NA	+Low	+Female	NS	+ Unemployed and change of job status	NS	NA	+	+
5	Middle	Gotsadze et al. (2009)	Georgia	NA	NA	NA	NA	+Low	NA	NA	NA	NA	+	NA	+
6	Middle	Somkotra, Lagrada (2009)	Thailand	NS	NS	+	NS	+ High	NS	NS	+Unemployed	+ Low	+	+	+
7	Middle	Shi et al. (2010)	China	NA	NA	NA	NA	+ Low	NA	NA	NA	NA	NA	NA	+
8	Middle	Mondal et al. (2010)	India	+	+Large	NA	NA	+Low	NS	NA	NA	NA	+	NA	+
9	Middle	Yardima, et al. (2010)	Turkey	+	NS	+	_	+Low	NS	NA	+Unemployed	+Low	NA	+	NA
10	Middle	Barros et al. (2011)	Brazil	NA	NA	+	NS	+ Low	+Female	NA	NA	NA	NA	NA	NA
11	Middle	Shi et al. (2011)	China	NA	+Small	+	NA	+ Low	NA	NA	+ Unemployed	NA	+	NA	+
12	Middle	Zhou, Gao (2011)	China	NS	+Small	+	NA	+ Low	NS	NA	NA	+ Low	+	NA	+
13	Middle	Amaya, Ruiz (2011)	Colombia	NA	+Small	NA	NA	+ Low	NS	+	+Self employed	NA	+	NS	NA
14	Middle	Pal (2012)	India	NA	+Large	+	+	NA	NS	+	NA	NS	NA	NA	NA
15	Middle	Li et al. (2012)	China	+	+Small	+	_	+ low	+Female	NA	+ Unemployed	+ Low	+	NA	+
16	Middle	Kavosi et al. (2012)	Iran	NA	NS	+	NS	+Low	NS	NA	NA	NA	+	+	NA
17	Middle	Moghadam et al. (2012)	Iran	NA	+Large	NA	NA	+Low	NA	NA	NA	NA	+	NA	NA
18	Middle	Chuma and Maina (2012)	Kenya	NA	NA	NA	NA	+Low	NA	NA	NA	NA	+	NA	NA
19	Middle	Arsenijevic et al. (2012)	Serbia	+	+Large	NA	NA	+Low	NS	NS	NS	+Low	NA	NA	+
20	Middle	Van Minh et al. (2013)	Bangladesh	NA	NA	NA	NA	+Low	NA	NA	NA	+Low	+	NA	+
21	Middle	Van Minh et al. (2013)	Viet Nam	+	−Large	+	+	+High	NS	NA	NA	NA	NA	NA	NA
22	Middle	Weraphong et al. (2013)	Thailand	NA	NA	NA	NA	+Low	NA	NA	NA	NA	NA	NA	NA
23	Middle	Li et al. (2013)	China	+	+Large	+	+	+Low	NA	NA	NA	+Low	+	NA	+
24	Middle	Misra et al. (2013)	India	NA	NS	NA	NA	NA	NA	NA	NA	NA	+	NA	NA
25	Middle	Ashour et al. (2013)	West Bank and Gaza (Palestine)	+	NA	NA	NA	+	+Female	NA	+ Unemployed	+Low	NA	NA	AN
26	Middle	Li et al. (2014)	China	NA	− Large	+	+	− Middle	+ Female	NA	+ Unemployed	+Low	+	NA	+
27	Middle	Narci et al. (2014)	Turkey	−Urban	−Large	+	+	+High	+ Female	NA	+ Unemployed	−High	NA	+	+
28	Middle	Brown et al. (2014)	Turkey	+	+Large	+	+	+High	+ Female	NA	NS	+Low	+	+	NA
29	Middle	Boing (2014)	Brazil	NA	NA	NA	NA	+Low	NA	NA	NA	+Low	NA	NA	NA
30	Middle	Khaing (2015	Myanmar	NS	+Large	NA	NA	NA	NS	NS	NA	+Medium	+	NA	NA
31	Middle	Htet (2015)	Myanmar	−Rural	+Large	+	+	+Low	+Female	NA	NA	NA	NA	NA	+
32	Middle	Buigut (2015)	Kenya	NA	NA	NA	NS	+High	NS	+	+ Unemployed	NA	NA	NA	NA
33	Middle	Xu (2015)	China	NA	+Small	+	NS	+Low	NS	NA	NA	NS	+	NA	+
34	Middle	Piroozi (2016)	Iran	NA	NS	+	NS	+Low	+Female	NA	NA	NA	+	+	NA
35	Middle	Masiye (2016)	Zambia	NS	NA	NA	NA	+Low	NS	+	NS	NS	NA	NA	NA
36	Low	Su et al. (2006)	Burkina Faso	NS	+Large	NA	NA	+Low	NS	NA	NA	NS	NA	NS	+
37	Low	Xu et al. (2006)	Uganda	+	NA	+	NA	NA	NS (among poor) +Female (non poor)	NA	NA	+Low	+	NA	NA
38	Low	Brinda et al. (2014)	Tanzania	NA	+Large	NA	NA	NA	NS	NS	+ Unemployed	NS	NA	+	+

NA (Non applicable)

NS (Not significant)

+ (Risk factor)

− (Protective factor)

**Table 3:** The role of the insurance in incurring CHE

Income category	Country	Insurance role
Middle	China (2010)	Significantly reduce CHE
Middle	Turkey (2010)	Significantly reduce CHE
Middle	Brazil (2011)	Risk factor
Middle	China (2011a)	Not significant
Middle	China (2011b)	Risk factor
Middle	Colombia (2011)	Significantly reduce CHE
Middle	China (2012)	Significantly reduce CHE
Middle	Iran (2012a)	Significantly reduce CHE
Middle	Iran (2012b)	Not significant
Middle	Vietnam (2013)	Significantly reduce CHE
Middle	Thailand (2013)	Not significant
Middle	China (2013)	Depends on the type of insurance scheme
Middle	West bank and Gaza (Palestine) (2013)	Significantly reduce CHE
Middle	China (2014)	Depends on the type of insurance scheme
Middle	Turkey (2014a)	Significantly reduce CHE
Middle	Turkey (2014b)	Significantly reduce CHE
High	South Korea (2016)	Not significant
